# Is less readable liked better? The case of font readability in poetry appreciation

**DOI:** 10.1371/journal.pone.0225757

**Published:** 2019-12-13

**Authors:** Xin Gao, Jeroen Dera, Annabel D. Nijhof, Roel M. Willems

**Affiliations:** 1 Centre for Language Studies, Radboud University, Nijmegen, The Netherlands; 2 Marketing and Consumer Behavior group, Wageningen University, Wageningen, The Netherlands; 3 Faculty of Arts, Department of Dutch Language and Culture, Radboud University, Nijmegen, The Netherlands; 4 King’s College London, London, United Kingdom; 5 Donders Institute for Brain, Cognition, and Behaviour, Radboud University, Nijmegen, The Netherlands; 6 Max Planck Institute for Psycholinguistics, Nijmegen, The Netherlands; University of Oslo, NORWAY

## Abstract

Previous research shows conflicting findings for the effect of font readability on comprehension and memory for language. It has been found that—perhaps counterintuitively–a hard to read font can be beneficial for language comprehension, especially for difficult language. Here we test how font readability influences the subjective experience of poetry reading. In three experiments we tested the influence of poem difficulty and font readability on the subjective experience of poems. We specifically predicted that font readability would have opposite effects on the subjective experience of easy versus difficult poems. Participants read poems which could be more or less difficult in terms of conceptual or structural aspects, and which were presented in a font that was either easy or more difficult to read. Participants read existing poems and subsequently rated their subjective experience (measured through four dependent variables: overall liking, perceived flow of the poem, perceived topic clarity, and perceived structure). In line with previous literature we observed a Poem Difficulty x Font Readability interaction effect for subjective measures of poetry reading. We found that participants rated easy poems as nicer when presented in an easy to read font, as compared to when presented in a hard to read font. Despite the presence of the interaction effect, we did not observe the predicted opposite effect for more difficult poems. We conclude that font readability can influence reading of easy and more difficult poems differentially, with strongest effects for easy poems.

## Introduction

There is a large body of literature that links *fluency* in cognitive processing to increased liking. Put simply, fluency theory predicts that things which are more easily (more fluently) perceived or understood, are liked better [1]. Fluency is prominently visible in canonical forms of poetry. Consider a sonnet: sonnets tend to have *meter* (a regular pattern of stressed and unstressed syllables), *rhyme* (the final words of lines end in the same phonological form), and a fixed length in terms of *stanzas*. Forms like that of a sonnet create high levels of expectation in readers and can be considered prime examples of language that can be processed fluently (fluency in this description pertains to form, it says nothing about how easily the content of the poem can be understood; we get back to this issue below). Empirical evidence indeed shows that increased fluency in the sense of the presence of rhyme or meter leads to increased memory or general appreciation of poetry [2–7]. The main thesis of fluency theory is that certain stimulus characteristics (e.g. rhyme, meter) increase *processing fluency*, that is, they increase the ease of processing of the stimulus. We call stimuli with such characteristics fluent stimuli.

It has been argued that ‘fluency’ is not one of a kind. Many scholars have distinguished at least between Perceptual and Conceptual fluency [1]. Perceptual fluency relates to how fluent the *perceptual* features of a stimulus are (e.g. symmetry, figure-ground segregation), conceptual fluency relates to how fluently the *meaning* of a stimulus can be understood. For instance, in the work by Menninghaus and colleagues [5], a distinction is made between perceptual (‘prosodic’) fluency and conceptual (‘semantic’) fluency in the processing of German proverbs. In a series of experiments, it was shown that beauty ratings of German proverbs were enhanced by the presence of rhetorical features such as rhyme. However, comprehensibility was decreased in the presence of rhetorical features. In the present study we will refer to ‘perceptual fluency’ as ‘structural fluency’ since it better captures the differences between the poems that we use as stimuli. It is worth noting that there is an alternative view in the literature on fluency which on the contrary states that all manipulations of fluency will have a similar, general effect. Alter and Oppenheimer for instance call fluency a ‘general metacognitive cue’ influencing preference and choice in various ways ([8], p. 220). In our research design we allow for the possible presence of differential effects of perceptual as well as structural fluency, following the findings cited above.

Specifically we hypothesized that structural and conceptual fluency would impact distinguishable aspects of language processing. In the present experiment we took this into account by having our experimental materials (the poems) differ in structural fluency (rhyme, meter), and conceptual fluency (clearness of topic). Moreover, we asked participants to rate the poems on both structural fluency (e.g. ‘flow’ of the poem; structural clarity) and conceptual fluency (e.g. topic clarity). There are arguably other characteristics in which poems can differ that impact conceptual or structural fluency, and the list of characteristics that we used was not meant to be exhaustive. Inspired by the different kinds of fluency, we chose to measure the poetry reading experience with four dependent variables. The first dependent variable was General Liking and was included to be an overall indicator of how much a reader liked poem. The second and third dependent variables were included to capture how structurally fluent a poem was perceived to be (‘Perceived flow’, and ‘Perceived Structure Clarity’). The fourth dependent variable was included to capture perceived conceptual fluency (‘Perceived Topic Clarity’).

Despite the fact that fluency is often found to be beneficial for understanding or appreciation of a stimulus, there is an interesting body of research showing on the contrary sometimes *disfluency* can enhance appreciation. Indeed, there is evidence showing that less fluent processing can make participants appreciate a stimulus more (see overview in 9). Clearly this is a counterintuitive finding if we consider the starting point of fluency theory, that is, that fluent stimuli will be liked more than disfluent stimuli. Alter [9] explains the potential benefits of disfluency as a processing advantage. The hypothesis is that disfluency allows for information to be processed more carefully and more deeply as compared to fluent information. Put differently, disfluency creates a ‘mental roadblock’ [9], meaning that disrupting the ease of processing intensifies the depth of cognitive processing. This increased depth of processing may heighten appreciation for specific stimuli, but decrease it for others, thus explaining the mixed findings. Typically, stimuli that benefit from disfluent processing are ‘difficult’ stimuli. An interesting related finding comes from Blohm and colleagues who showed that grammaticality judgements and poeticity judgements of the same sentences led to opposite effects [10]. Sentences that score low on grammatical acceptability scored high on poeticity and vice versa. This is an illustration of how disfluency in one domain (grammar) can lead to increased subjective rating in another domain (poeticity).

An example of the benefits of disfluency which is particularly relevant for the present paper is the finding that the Moses illusion is influenced by font readability. The Moses illusion is the classical psycholinguistic observation that large proportions of participants tend to overlook a factual error when that error occurs in a situation with a strong semantic attraction. In the original experiment Erickson and Mattson [11] asked participants how many exemplars of each species Moses took on board of his arc. The participants–when asked explicitly–knew that it was not Moses but Noah who built an arc in the canonical Biblical narrative. However, in their initial reading, a substantial proportion of participants overlooked the erroneous mentioning of Moses. One explanation is that the semantic attraction between Moses and Noah is so strong that writing ‘Moses’, when ‘Noah’ is meant, creates a semantic illusion (see e.g., [12–14]). Interestingly, Song and Schwarz showed that changing the readability of the font of the question influenced the proportion of participants that noticed the error [15]. That is, when the font was literally more difficult to read, participants became better at spotting the error, making the semantic illusion less strong (see [16,17,18] for related examples; see [19,20] for adverse findings). The authors explain the beneficial effect of decreased readability as a reflection of increased processing resources that are devoted to the sentence that participants read. The experiment by Song and Schwarz is an example of how less fluent processing can lead to deeper cognitive processing (see [9] for comprehensive review).

It is interesting to note that poets and poetry publishers have since long experimented deliberately with presenting poems in different fonts. Typographic manipulations play a central role in the poetics of, for instance, Concrete Poetry [21] and Dadaism [22], while changes in font have occurred throughout literary history from Antiquity onwards [23]. In the case of modern Dutch poetry, which was used in the experiments reported in this article, the semantic effects of font manipulation as poetic device have been analyzed for various authors, such as the early 20^th^-century avant-gardist Paul Van Ostaijen [24], *De Stijl*-artists like Theo van Doesburg and Piet Mondriaan [25], and the postmodern poet Tonnus Oosterhoff [26]. Experiments with poetry books as objects, including their typographic and font designs, are also common in contemporary poetry aesthetics [27,28].

In processing models of literary reading, there is an interesting conceptual parallel with the fluency–disfluency distinction as introduced above. It can be argued that the equivalents of processing fluency and disfluency in literary reading are *backgrounding* and *foregrounding*. During literary reading there is a proposed optimal balance between content which is predictable and can be processed fluently (backgrounded information), and content which stands out in terms of language use (foregrounded information). Jacobs incorporates the effects upon the readers of both text aspects in his Neurocognitive Model of Literary Reading [29,30]. The model predicts that it is especially foregrounding which promotes aesthetic appreciation of language / literature, and asserts that slower reading is part of noticing foregrounding during reading ([30]). This is nicely in line with the suggested deeper processing resulting from disfluency (‘foregrounding’ [31]) as described above. The overlap between these concepts is certainly not one-to-one, but they seem related enough to deserve explicit mentioning.

Conceptually we highlight the overlap between fluency theory and the theory on foregrounding versus backgrounding as an example of how our study relates to somewhat separated research traditions. We believe that uniting a fluency perspective with foregrounding-backgrounding could be a fruitful avenue for research. For fluency researchers this is relevant since it could open up the study of a phenomenon–poetry–which is known to rely heavily on fluency. For literary scholars it opens the possibility of connecting with a rich psychological literature, including several processing models (see above). By no means do we want to claim to be original in combining fluency theory with poetry reception research, we are merely pointing out the potential of the combination for the traditionally separated readerships.

In conclusion, both fluency and disfluency can lead to increased appreciation of (literary) language. Moreover, deeper processing has been associated with increased noticing of foregrounded passages in literary text. Combined with the work reviewed above we predict that poem difficulty and font readability will interact in the present experiment. We ask whether poems that are high or low in fluency are appreciated better depending on the readability of the font they are presented in. We investigated this in three separate experiments in which a wide range of participants (N = 513) read original Dutch poems. We corrected our results for overall differences in age and self-reported general liking of poetry, since these are factors irrelevant to the research question, but that will most likely influence the scorings that participants give.

In our design we manipulate Font Readability (an implementation of processing fluency at a basic level), and Poem Difficulty (a combination of structural and/or conceptual fluency, depending on the experiment). We did this in three separate experiments, using different poems which had higher or lower conceptual and structural fluency. Our first hypothesis results from our main research question: Font readability will differentially affect appreciation (measured in different ways) of easy and difficult poems. For easy poems, a less readable font will lead to lower appreciation. This prediction follows the basic fluency theory claim that a decrease in processing fluency leads to lower appreciation [32]. On the contrary, we expect that for difficult poems the less readable font will lead to higher appreciation. That is, the break in reading fluency (because of the difficult font) may increase attention and / or processing time devoted to the difficult poems, leading to a higher subjective rating. Statistically this hypothesis would be visible as a Font Readability x Poem Difficulty interaction effect.

Our second hypothesis stems from the conceptual versus structural fluency distinction. The interaction effect between Font Readability and Poem Difficulty would be different for perceived structural and conceptual fluency across the three experiments. Structural and conceptual fluency were measured with distinct dependent variables. Our prediction therefore was that the effects of a Font Readability x Poem Difficulty interaction would be present on some dependent variables in a particular experiment only. After all each experiment used poems that scored higher or lower on structural or conceptual fluency. Alternatively, if every source of fluency influences processing in a similar manner [8,33], we would not expect differences in conceptual or structural fluency to lead to different effects across experiments.

## Methods

### Materials

The materials used in the three experiments consisted of six (2 per experiment) short poems (twenty verse lines or less) by Dutch authors, all of whom publish their poetry with well-regarded literary publishers. The poems differed in both structural fluency and conceptual fluency (see Table 1). Note that all of our materials were original, unmodified poems from Dutch poets. The poems are diverse, and they are not classifiable as falling within a common stylistic classification. All poems were modern (five were published between 1987–2013, one in 1934). A loose selection criterion that we had is that they should be ‘readable’ by the diverse and modern readership that formed our subject sample. Another selection criterion was that the poems should not be very long for reasons of experimental feasibility. The number of words ranged between 55–123, with a median of 102.5 (see Table 1).

**Table 1:** Characterization of the poems that were used as stimulus materials. Each poem was scored on aspects related to structural or conceptual fluency. See the text for a detailed description of how the scoring was done. Gramm. = Grammaticality.

Title	Poet (Year)	Rhyme	Meter	Gramm.	Structure markers	Ambiguity (reversed)	Topical sentences	Total structural clarity	Total conceptual clarity	Condition
Sterfbed	Jean-Pierre Rawie (1992)	3	3	3	3	3	3	12	6	EASY
Alles gefilmd	Robert Anker (1987)	2	2	2	1	1	1	7	2	EASY
Het kind en ik	Martinus Nijhoff (1934)	3	3	3	3	1	1	12	2	EASY
En omgekeerd	Rutger Kopland (2001)	1	1	3	3	2	2	8	4	HARD
Meisje in het najaar	Ingmar Heytze (1997)	1	2	3	2	3	1	8	4	HARD
Wanhoop als een kuil afschilderen	Vicky Francken (2013)	2	1	2	1	1	1	6	2	HARD

The poems were each rated on their structural and conceptual difficulty by a poetry expert (one of the authors, JD). Importantly, the rater was naïve as to the purpose of the experiment when he rated the poems. With regard to the structural components, the poems were scored either ‘low’, ‘medium’ or ‘high’ on the following parameters:

- *Rhyme*: ‘low’ in the case of poems that show no (or very limited) examples of sonic foregrounding through similar sounds in stressed syllables; ‘medium’ in the case of poems that do show end rhyme, but not as an organizing principle; ‘high’ in the case of poems that have end rhyme as a structural principle.
- *Meter*: ‘low’ in the case of poems that consist of free verse; ‘medium’ in the case of poems that lack a consistent metrical scheme throughout the whole poem, but do show a regular distribution of syllables within specific stanzas or groups of verse lines; ‘high’ in the case of metrical poems that use a regular distribution of stressed syllables in each verse line.
- *Grammaticality*: ‘low’ when a considerable amount (>20%) of the poem’s sentences infringe on grammatical rules; ‘medium’ when this is the case in a limited number of phrases (5–20%); ‘high’ in the case of poems that do not (or hardly) infringe on grammar (<5%).
- *Structure markers*: ‘low’ in the case of poems that do not make use of conjunctions and transitional phrases to connect verse lines; ‘medium’ when these types of signal words occur in a limited number of verse lines (1–20% of sentences); ‘high’ in the case of poems that contain many conjunctions and transitional phrases (>20% of sentences).

With regard to conceptual clarity, the poems were scored on two parameters:

- *Ambiguity*: ‘low’ in the case of poems that communicate their message directly; ‘medium’ when a limited number of verse lines can be interpreted in multiple ways; ‘high’ in the case of poems that undermine direct communication by constructing multiple layers or even cryptic, hermetic verse lines.
- *Topical sentences*: ‘low’ in the case of poems that do not emphasize their own theme or express the explicit emotions of a lyrical I-figure; ‘medium’ in the case of poems that contain a single verse line that summarizes the point made by the author; ‘high’ when the poet repeatedly mentions his/her theme or emotions.

We note that delineating the criteria as instances of ‘structural’ or ‘conceptual’ fluency is most likely to be a matter of degree. The structural components are mostly structural and the conceptual components as best characterized as conceptual. We do not mean to imply that either is a ‘pure’ case of structural or conceptual difficulty. Table 1 displays these verbal descriptions as scores. A score of ‘3’ is given for a property which makes the poem score high on structural or conceptual fluency (e.g. ‘high’ score on rhyme), ‘2’ denotes medium fluency, and ‘1’ scores low fluency. We now describe each poem’s structural and conceptual clarity in turn.

The first poem (experiment 1) is ‘Sterfbed’ (‘Deathbed’) by Jean-Pierre Rawie. It scores very high on structural fluency: it contains both end rhyme and iambic pentameter as structural principles; it does not violate Dutch syntactic rules; each stanza contains several conjunctions and there are no metaphors that its readers need to decipher. The conceptual fluency of this poem is equally high: there are no ambiguous verse lines and many topical sentences.

The second poem (experiment 1), ‘Alles gefilmd’ (‘All filmed’) by Robert Anker, on the other hand, scores relatively low on structural fluency. It does not make use of rhyme as an organizing principle, although some verse lines show equivalence through the use of assonance in the end words (‘Op een ochtend als de wereld overloopt’ / ‘en schiet alle bloemen bij de buren alle dood’ + ‘Alles gefilmd door de media. De bloemen,’ / ‘de emoties in de buurt, kijk, zijn schoenen’). Also, the poem is definitely rhythmical and the syllables are distributed relatively equally along the verse lines, but it lacks a regular metrical scheme. Two phrases in the poem infringe on the grammatical rules of Dutch, one of them being an ellipsis (‘Kan deze nieuwe wijk een nest tegen de wereld’) and the other an anacoluthon (‘Op een ochtend als de wereld overloopt, / dat hij dan de ramen openzet’). Furthermore, the poem contains very little conjunctions and transitional phrases while it contains many metaphors. As a consequence, its conceptual fluency is low: there are many ambiguous phrases and little topical sentences.

The third poem, ‘Het kind en ik’ (‘The child and I’) by Martinus Nijhoff (experiment 2), scores high on structural fluency, but low on conceptual clarity. The poem contains end rhyme as an organizing principle and although there is no regular metrical scheme, Nijhoff distributes his syllables relatively equally along the verse lines. Apart from the position of the direct object in the fourth stanza (‘al wat ik van mijn leven / nog ooit te schrijven droom’), the poem does not violate Dutch syntax, while it also facilitates the reading experience by making use of transitional phrases (‘Maar’, ‘en’). The poem is high in metaphor density: in fact, the poem itself *is* a metaphor. Hence, the conceptual fluency of the poem is relatively low. Not only is it highly ambiguous, it also lacks topical sentences that emphasize its actual theme.

The fourth poem, ‘En omgekeerd’ (‘And in reverse’) by Rutger Kopland (experiment 2), shows a rather complex pattern. For there is no meter involved and little rhyme, the poem does not facilitate structural fluency on a phonological level. Grammatically, though, the poem is fluent, while it is high in conjunctions and low in metaphors. Still, there are some ambiguous lines in the poem and although the poet concludes with a rather topical stanza, its conceptual clarity is certainly not high.

The fifth poem, ‘Meisje in het najaar’ (‘Girl in autumn’) by Ingmar Heytze (experiment 3), shows a rather equal pattern with regards to its structural fluency. Both rhyme and meter are relatively absent, but the poet does not infringe on grammar. Also, Heytze subtly makes use of antonyms in order to structure his stanzas and is spare with complex metaphors. The ambiguity of this poem, then, is relatively low. Yet, there are no topical sentences that explicitly underline the poem’s theme.

The final poem, ‘Wanhoop als een kuil afschilderen’ (‘To depict despair like a pit’) by Vicky Francken (experiment 3), scores low on both structural and conceptual clarity. Its free verse is not organized by rhyme. The poem does not radically infringe on Dutch grammar, but its sentence structure is elliptical from the start. Also, Francken hardly makes use of conjunctions and transitional phrases, while the metaphor density of the poem is high. Hence, the poem contains many ambiguous phrases and scores low on topical sentences.

In sum, Table 1 shows that the ‘Easy’ poems scored higher on structural and / or conceptual fluency, although the match between poems was not always perfect (e.g. in experiment 2, the ‘Easy’ poem scores lower on conceptual fluency as compared to the ‘Hard’ poem). Inspection of the table shows that in Experiments 1 and 2, the poems mainly differed in terms of structural fluency, whereas in Experiment 3 conceptual fluency was most different between the poems. Note that the distinction between Easy and Hard poems is made within each experiment. Put differently, it is not an absolute score: the Easy poem in Experiment 3 had a similar fluency rating as the Hard poem of Experiment 2.

**Fig 1:**
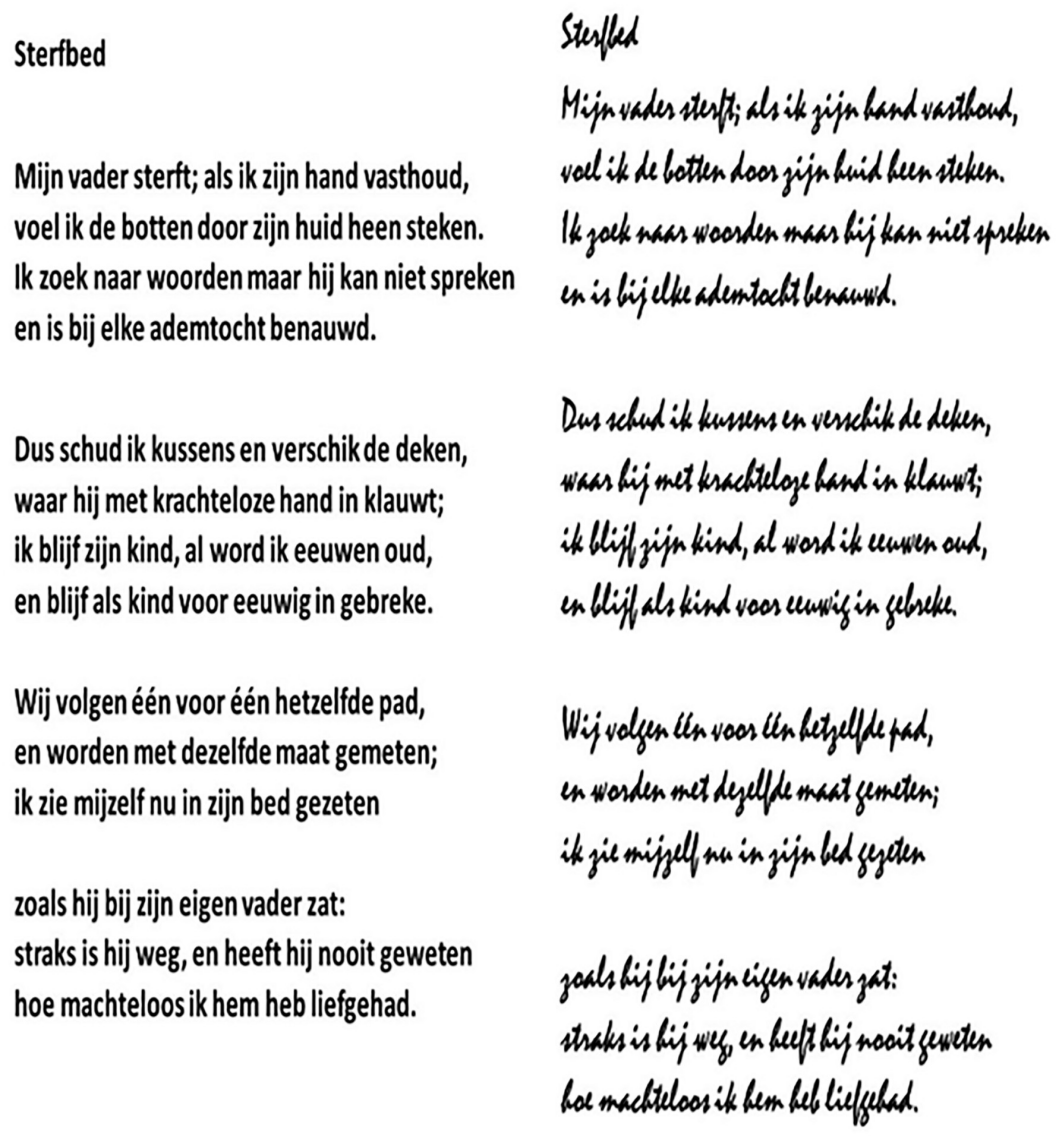
Example of stimuli used. Displayed is the poem ‘Sterfbed’, an Easy poem in terms of fluency (Table 1), in the two fonts. On the left is the more readable font (Calibri) and on the right is the less readable font (Mistral).

To manipulate Font Readability, two fonts were used (Fig 1). As readable font (High on Readability), we used font Calibri (This is an example), and as less readable font we used Mistral (This is an example). Fig 1 shows the same poem printed in both fonts. The readability of the fonts was tested within the present experiments by adding a font readability control question to the questionnaire. To match the size of letters on screen or on printed pages, font size was 15 (Calibri) or 20 (Mistral) points.

### Experimental procedure

Experiment 1 was partially done on-line (in the ‘Qualtrics’ testing environment), and partially in the lab. Experiments 2 and 3 were audience participation experiments, performed during a music festival (Experiment 2, ‘Music meeting’, June 5 2017, Nijmegen) and during a theatre / food festival (Experiment 3, ‘Festival op ‘t Eiland’, July 16 2017, Nijmegen). In Experiment 1 participants filled out the questionnaire online or performed it in a behavioral testing lab for course credit or monetary reward at the Centre for Language Studies, Radboud University, Nijmegen. Advertisement for the on-line version of the experiment was done via social media. In Experiments 2 and 3 visitors of the two festivals were asked to participate. No reward was given for participation during the festivals. All materials are available on the open science framework (OSF) project page (https://osf.io/jwcqt/).

Participation in the experiment was easy and consisted of working through three pages. The first page was a welcome page. It instructed participants that they would be reading a poem and that they would be asked questions about the poem afterwards. It emphasized that there were no right or wrong answers. Participants were instructed to read the poem as they usually would. On page 2 the poem was printed (in one out of four conditions, see below). Page 3 was the questionnaire. Participants answered 8 questions about the poem (Table 2). Finally, they filled out 4 additional questions: Gender (female, male, other), age, ‘On average, how often do you read poetry?’ (4 response options), ‘do you consider yourself a poetry lover?’ (7 point Likert scale). The questionnaire can be found on the OSF project page (https://osf.io/jwcqt/).

**Table 2:** Items of the questionnaire. The first four items were used as dependent variables in the analysis. Items 5 and 6 were control items, and items 7 and 8 assessed whether participants already knew the poems. Likert scales ranged from ‘Not at all’ (1) to ‘Very much’ (7).

Number	Question		Response options	Label
	Dutch	English translation		
1.	Ik vond dit een mooi gedicht	I liked this poem	7-point Likert	General appreciation
2.	Ik vond het gedicht ‘lekker lopen’	I thought the poem had a nice flow	7-point Likert	Perceived Flow
3.	Het gedicht had een duidelijke structuur	The poem had a clear structure	7-point Likert	Perceived Structure Clarity
4.	Het onderwerp van dit gedicht was duidelijk	The topic of the poem was clear	7-point Likert	Perceived Topic Clarity
5. Exp1	Ik vond het gedicht emotioneel	I thought the poem was emotional	7-point Likert	Emotionality (not used in analysis)
5. Exp 2 and 3	Ik vond het lettertype prettig leesbaar	I thought the font was nice to read	7-point Likert	Font Readability (control item)
6.	Ik vond het een moeilijk gedicht	I thought this was a difficult poem	7-point Likert	Poem difficulty (control item)
7.	Kende u het gedicht?	Did you know the poem?	No / Yes / I’m not sure, maybe	Recognize (exclusion criterion)
8.	Weet u wie het gedicht geschreven heeft?	Do you know who wrote the poem?	No / Yes, [open response option]	Author
9.	Beschouwt u zichzelf als een poeziëliefhebber?	Do you consider yourself to be a poetry lover?	7-point Likert	Poetry Lover

Four dependent variables were used in the main analysis (see Table 2). They tested General Appreciation (questionnaire item 1), Perceived Flow (item 2), Perceived structure (item 3), Perceived Topic clearness (item 4). Item 5 measured how emotional the poem was perceived to be in Experiment 1, but was changed to a control variable in experiments 2 and 3, measuring the readability of the Font. Item 6 measured perceived difficulty and was added as a manipulation check. Items 7 and 8 tested whether participants already knew the poem before taking part in the experiment. These items were to measure whether our participants were naïve with respect to the materials or not.

### Preregistration and sampling plan

We pre-registered planned sample sizes, experimental design, hypotheses, analyses plans on the Open Science Framework (https://osf.io/hc3xe/ and https://osf.io/fuen8/). Since we initially planned to perform only one experiment, analyses are different from the preregistration in the sense that the factor Experiment was added to the ANCOVAs (see below). The study was approved by the institutional ethics review board of the Centre for Language Studies, Radboud University.

Sample size was established in a sequential testing procedure with (*f* = 0.14) as the smallest effect size of interest (SESOI) [34]. This effect size was based on the willingness to collect maximum 400 participants for each study, in order to achieve 80% power and 5% of alpha level (G*Power). We decided to perform two-sided interim analysis twice, respectively after collecting 200 and 400 participants. The alpha boundaries for the two analyses were calculated by WinLD software. In this study, we mainly focused on the interaction effect between font readability and poem fluency. Thus, we compared p-value and effect size of the interaction to the adjusted alpha level and SESOI. For experiments 1 and 2 we followed the sampling plan as just described. For experiment 3 we did not pre-specify the sample size. In total 541 participants took part in the experiments (N_exp1_ = 200; N_exp2_ = 200; N_exp3_ = 141).

### Participants

In total 541 participants took part in the study, distributed over 3 separate experiments (Exp. 1: N = 200; Exp. 2: N = 200; Exp. 3: N = 141). Informed consent was obtained verbally. The data from participants that indicated to already know the poem were removed (19 participants). Responses from participants younger than 18 years (9 participants) were also removed. It was not planned to test minors, but in the process of testing some minors were very eager to participate, for instance because their parents did. In such cases consent was asked from the parents and we had minors fill in the questionnaire. This resulted in the data from N = 513 participants entering the final analysis. Mean age was 37.13 years (median = 33; range 18–75, SD = 15.14, IQR = 27.25). In total 303 participants were female, 206 were male, and 4 participants indicated their gender as unspecified. The distribution across the conditions was as follows: Easy Poem, Easy Font N = 131; Easy Poem, Difficult Font N = 136; Difficult Poem, Easy Font N = 122; Difficult Poem, Difficult Font N = 124.

In Experiment 1, 66 participants performed the experiment online via the Qualtrics survey software. The remaining participants were tested in a psychological laboratory environment on the Radboud University campus. In total N = 186 participants’ data were entered into the main analysis from experiment 1 (128 female, 55 male, 3 unspecified; mean age = 25.26; median = 23, range = 18–65, SD = 8.12, IQR = 6).

From Experiment 2 the data from N = 187 participants were added to the analysis (97 female, 90 male, 0 unspecified; mean age = 45.25; median = 48.5, range = 18–75, SD = 15.50, IQR = 30).

From Experiment 3 the data from N = 140 participants were added to the analysis (78 female, 61 male, 1 unspecified; mean age = 41.42; median = 40, range = 18–70, SD = 11.79, IQR = 15).

### Experimental design and statistical analysis

The experimental design consisted of three factors, with either three or two levels (3x2x2 design). The factors were: Experiment (Experiment 1, Experiment 2, Experiment 3); Font Readability (Easy, Difficult); Poem Difficulty (Easy, Difficult). Statistical analysis was done using 3-factor between-subjects ANCOVAs. Next to the three factors of interest, as covariates we added responses to the question “how much do you consider yourself a poetry lover”, and Age. These covariates were added since we expected them to explain additional variance (e.g. [35,36]). Separate ANCOVAs were run for each of the four dependent variables: 1) General appreciation, 2) Perceived Flow, 3) Perceived Structure Clarity, and 4) Perceived Topic Clarity. All analyses were performed in the JASP statistical software package [37].

The main focus of analysis was the hypothesized Font Readability x Poem Difficulty interaction on each of the four dependent variables as explained above. Although the experimental design of all three studies was the same, because of the differences in conceptual and/or structural fluency in the poems we had reason to expect that the poems that were used in the respective experiments would elicit different effects in the font manipulation. For instance, the experiment with the highest difference between poems in conceptual fluency, would show the largest effect on the dependent variable measuring conceptual understanding. We therefore tested for the crucially expected Poem Difficulty x Font Readability in a 3-way between-subjects ANCOVA with factors Font Readability (Easy, Difficult), Poem Fluency (Easy, Difficult), Experiment (Exp 1, Exp 2, Exp 3).

## Results

All data and code to create the plots as reported in the paper are available on the open science framework (https://osf.io/jwcqt/).

The font readability control item (item 5 in Table 2) showed that the less readable fonts were considered less nice to read, as was to be expected (Mean Easy font = 2.10, SD = 1.11, Mean Difficult font = 5.88, SD = 1.21; t(325) = 29.48, *d* = 3.26, p<0.001).

The poem difficulty control item (item 6 in Table 2) showed that overall participants found the Hard poems more difficult than the Easy poems. Difficult poems were scored as more difficult than Easy poems (Across all experiments: Mean Difficult poem = 4.25, SD = 1.50, Mean Easy Poem = 3.15, SD = 1.51; t(510) = 8.28, p<0.001, *d* = 0.732; Exp. 1: Mean Difficult poem = 4.86, SD = 1.30, Mean Easy Poem = 2.92, SD = 1.24, t(183) = 10.36, p<0.001, *d* = 1.52; Exp. 2: Mean Difficult poem = 4.16, SD = 1.52, Mean Easy Poem = 3.43, SD = 1.62, t(185) = 3.17, p = 0.001, *d* = 0.46; Exp. 3: Mean Difficult poem = 3.57, SD = 1.62, Mean Easy Poem = 3.06, SD = 1.41, t(138) = 1.98, p = 0.025, *d* = 0.34).

For each of the four dependent variables there were large main effects of Poem Difficulty with participants favoring Easy over Difficult poems (Table 3A–3D).

**Table 3:** Results of the statistical analysis. ANCOVA’s were done for each of the four dependent variables General Liking, Perceived Flow, Perceived Topic Clarity, and Perceived Structure Clarity. The ANCOVA’s had three factors: Poem Difficulty (Low, High), Font Readability (Low, High) and Experiment (Exp. 1, Exp. 2, Exp. 3). The covariates Age and the answer to the question ‘Do you consider yourself to be a poetry lover?’ were added as covariates to explain additional variance. Note that for none of the dependent variables a statistically significant 3-way interaction was observed. We therefore did not analyze the data per experiment. We did observe a Poem Difficulty x Font Readability interaction for three of the four dependent variables, indicating that Font Readability has a differential effect on poems that have low or high difficulty.

**A. ANCOVA—General Liking**						
	**Sum of Squares**	**df**	**Mean Square**	**F**	**p**	**η**^**2**^_**p**_
Poem Difficulty	95.710	1	95.710	67.714	**< .001**	0.121
Font Readability	11.394	1	11.394	8.061	**0.005**	0.016
Experiment	1.116	2	0.558	0.395	0.674	0.002
Poem Difficulty * Font Readability	8.287	1	8.287	5.863	**0.016**	0.012
Poem Difficulty * Experiment	79.313	2	39.657	28.057	**< .001**	0.103
Font Readability * Experiment	2.784	2	1.392	0.985	0.374	0.004
Poem Difficulty * Font Readability * Experiment	3.818	2	1.909	1.351	0.260	0.005
Age	12.458	1	12.458	8.814	**0.003**	0.018
Poetry Lover	41.527	1	41.527	29.380	**< .001**	0.057
Residual	692.588	490	1.413			
**B. ANCOVA–Perceived Flow**						
	**Sum of Squares**	**df**	**Mean Square**	**F**	**p**	**η**^**2**^_**p**_
Poem Difficulty	102.599	1	102.599	64.620	**< .001**	0.117
Font Readability	3.084	1	3.084	1.943	0.164	0.004
Experiment	2.369	2	1.185	0.746	0.475	0.003
Poem Difficulty * Font Readability	9.140	1	9.140	5.757	**0.017**	0.012
Poem Difficulty * Experiment	116.590	2	58.295	36.716	**< .001**	0.130
Font Readability * Experiment	7.280	2	3.640	2.293	0.102	0.009
Poem Difficulty * Font Readability * Experiment	0.383	2	0.191	0.121	0.886	0.000
Age	42.778	1	42.778	26.943	**< .001**	0.052
Poetry Lover	46.246	1	46.246	29.127	**< .001**	0.056
Residual	777.988	490	1.588			
**C. ANCOVA–Perceived Structure Clarity**						
	**Sum of Squares**	**df**	**Mean Square**	**F**	**p**	**η**^**2**^_**p**_
Poem Difficulty	115.247	1	115.247	68.079	**< .001**	0.122
Font Readability	1.959	1	1.959	1.158	0.283	0.002
Experiment	5.264	2	2.632	1.555	0.212	0.006
Poem Difficulty * Font Readability	21.322	1	21.322	12.595	**< .001**	0.025
Poem Difficulty * Experiment	23.687	2	11.843	6.996	**0.001**	0.028
Font Readability * Experiment	4.043	2	2.021	1.194	0.304	0.005
Poem Difficulty * Font Readability * Experiment	5.270	2	2.635	1.556	0.212	0.006
Age	20.971	1	20.971	12.388	**< .001**	0.025
Poetry Lover	29.856	1	29.856	17.636	**< .001**	0.035
Residual	829.493	490	1.693			
**D. ANCOVA–Perceived Topic Clarity**						
	**Sum of Squares**	**df**	**Mean Square**	**F**	**p**	**η**^**2**^_**p**_
Poem Difficulty	179.113	1	179.113	93.235	**< .001**	0.160
Font Readability	0.407	1	0.407	0.212	0.646	0.000
Experiment	34.297	2	17.148	8.926	**< .001**	0.035
Poem Difficulty * Font Readability	4.770	1	4.770	2.483	0.116	0.005
Poem Difficulty * Experiment	139.762	2	69.881	36.376	**< .001**	0.129
Font Readability * Experiment	0.783	2	0.392	0.204	0.816	0.001
Poem Difficulty * Font Readability * Experiment	0.533	2	0.267	0.139	0.870	0.001
Age	26.652	1	26.652	13.873	**< .001**	0.028
Poetry Lover	24.579	1	24.579	12.794	**< .001**	0.025
Residual	941.335	490	1.921			

As concerns our main analysis, the ANCOVA showed that there was no statistically significant 3-way Poem Difficulty x Font Readability x Experiment interaction for any of the four dependent variables (Table 3A–3D). Therefore, we choose not to discuss results separately for the three experiments but to collapse all results over experiments instead.

**Fig 2:**
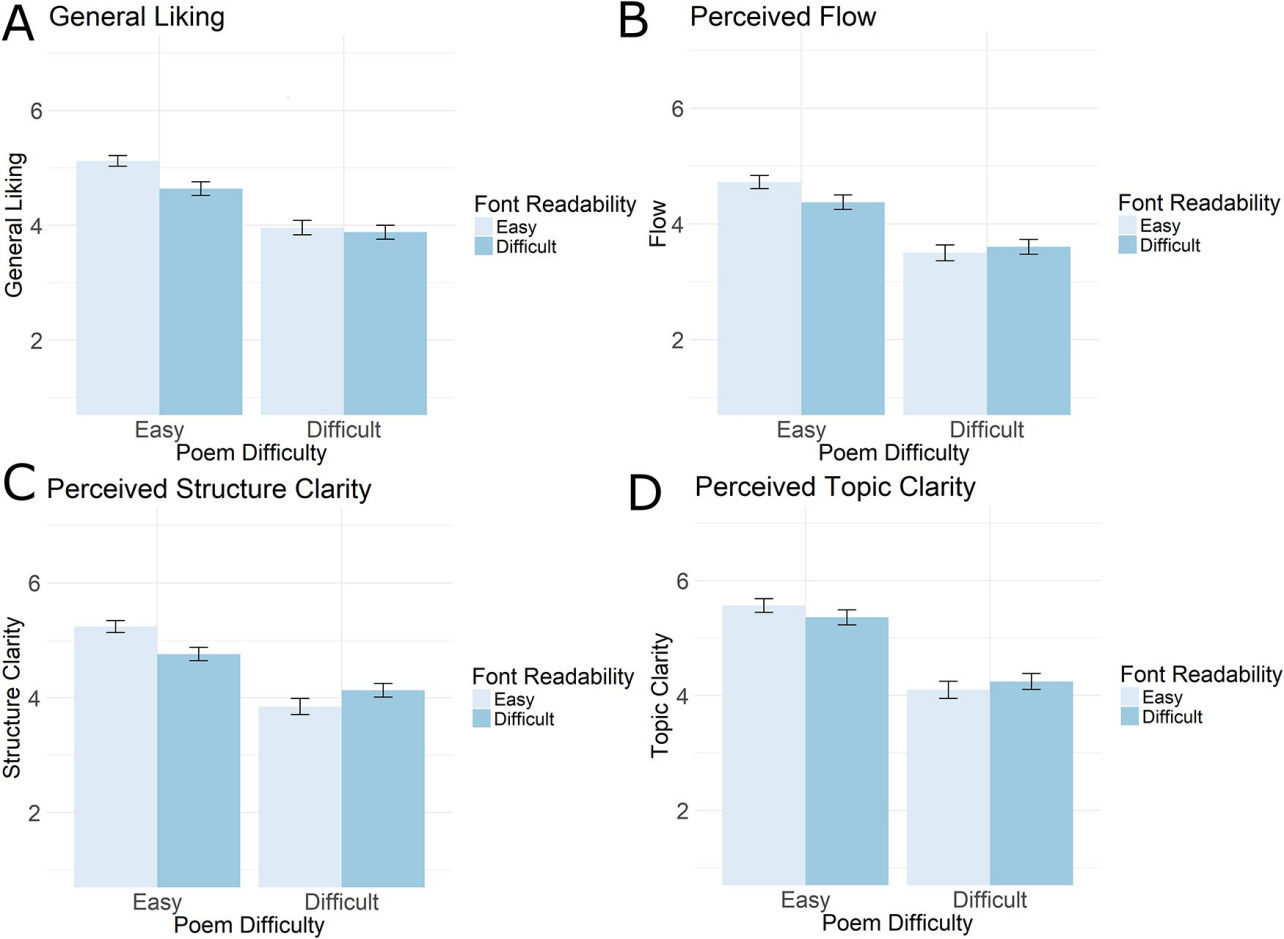
Illustration of results. Bar graphs display mean scores (all scored on 1–7 Likert scales) for Easy or Difficult Poems (x-axis) depending on the Readability of the Font they were presented in (light and dark blue fill). For the dependent variables General Liking (A), Perceived Flow (B), and Perceived Structure Clarity (C) we observed statistically significant interactions between Font Readability and Poem Difficulty. This means that font readability affected the reading experience of Easy and Difficulty poems in different ways. Easy poems are scored lower on these three dependent variables when they are presented in a harder to read font compared to when presented in the easier to read font (left two bars). For the difficult poems there is numerical trend towards the opposite effect (scored higher when presented in hard to read font, right two bars), but this was not statistically reliable (see Results section). No Font Readability x Poem Difficulty interaction was observed for the fourth dependent variable, Perceived Topic Clarity (D). Note that the pattern for each dependent variable looks comparable, which is to be expected given the sizeable correlations between the scores. See text for statistical tests between conditions. Error bars represent 95% confidence intervals.

**Fig 3:**
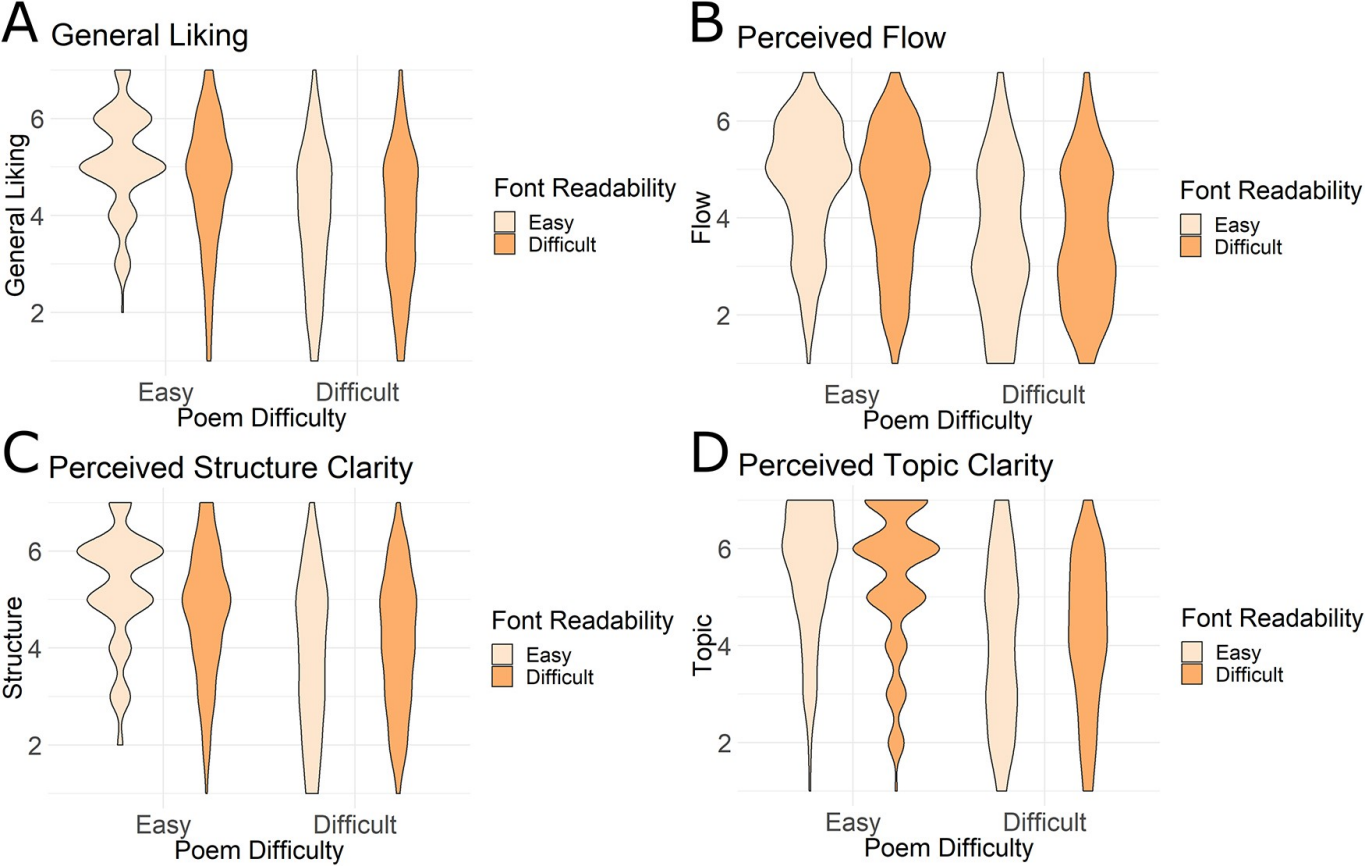
Same data as in Fig 2, now displayed as violin plots to show the distribution of the data. Note that the distribution of scores for Easy poems (left violins) are narrower than those for the Difficult poems (right violins). This shows that scoring was more similar across participants for the Easy as compared to the Difficult poems.

**Fig 4:**
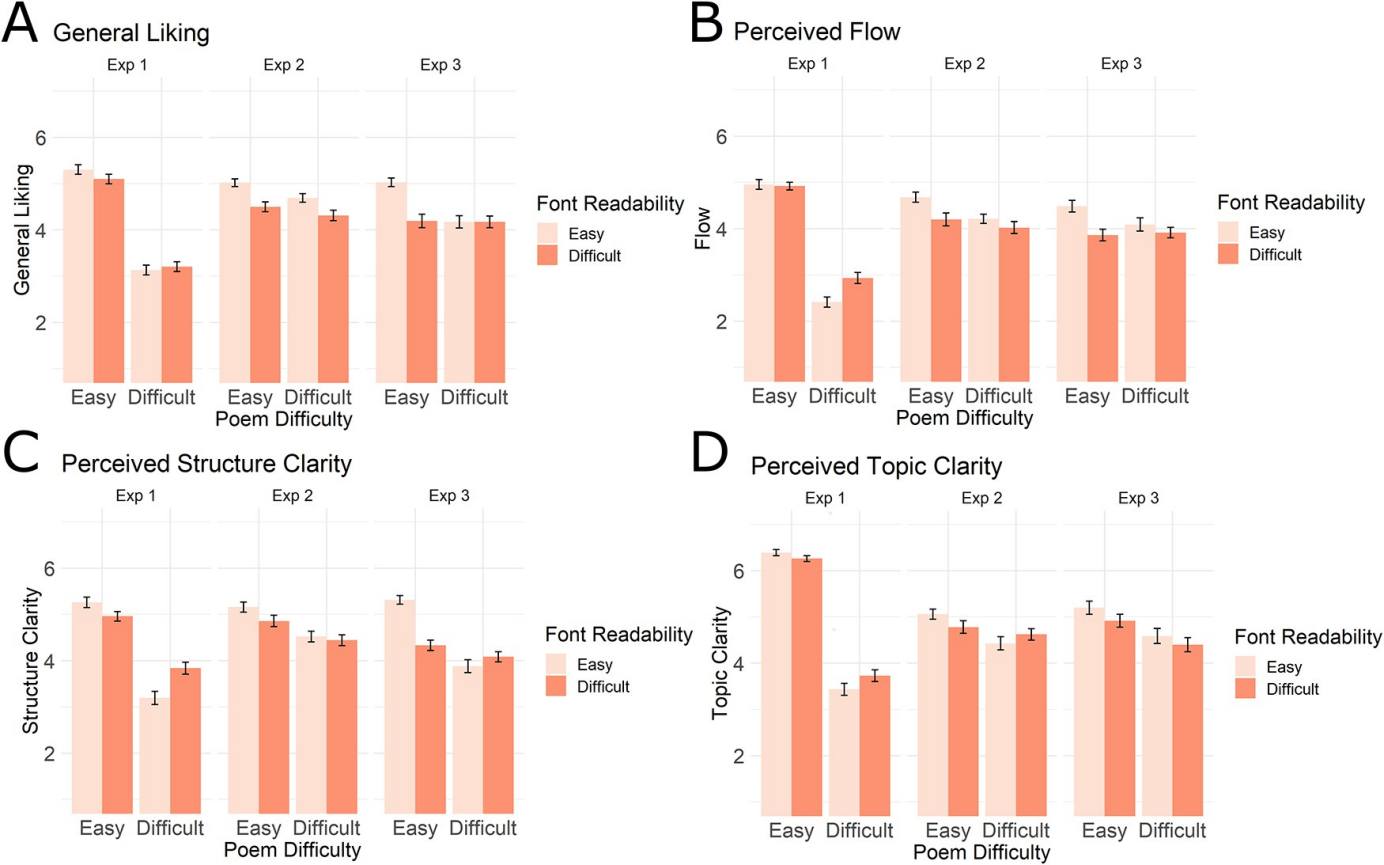
Bar graphs per experiment. Bar graphs display the mean scores (scorings on 1–7 Likert scale) for all conditions, for each Experiment separately. Note that different participants took part in each experiment and that different poems were used per experiment. Since the factor Experiment did not interact statistically with any other factor, we decided to collapse over Experiment in the results (see Fig 2). To give a complete picture of the data, we additionally plot the results per experiment here. Error bars represent 95% confidence intervals.

All results for the first dependent variable (‘General Liking’) are presented in Table 3A and are illustrated in Figs 2A, 3A and 4A. We observed that easy poems were liked better than more difficult poems (main effect of Poem Difficulty) and that poems presented in the easy to read font were liked better than poems presented in the difficult to read font (main effect of Font Readability). Crucially, there was a statistically significant Poem Difficulty x Font Readability interaction (Table 3A and Fig 2A). Font Readability did have an influence on General Liking of the Easy poems (Easy poem, Easy versus Difficult font: M_difference_ = 0.48, t(265) = 3.251, Cohen’s *d* = 0.40, p = 0.002), but not on the difficult poem (Difficult poem, Easy versus Difficult font: M_difference_ = 0.08, t(244) = 0.449, *d* = 0.06, p = 0.654). This suggests that a break in readability (decreased processing fluency) makes participants like a poem less when the poem is conceptually easy to understand, but not when it is more difficult to understand.

All results for the second dependent variable (‘Perceived Flow’) are presented in Table 3B and illustrated in Figs 2B, 3B and 4B. We observed that the Flow of easy poems was judged higher as compared to more difficult poems (main effect of Poem Difficulty). Again, and crucially, there was a statistically significant Poem Difficulty x Font Readability interaction. As was the case for General Liking, the Easy poems are most affected by a difference in Font Readability (Easy poem, Easy versus Difficult font: M_difference_ = 0.35, t(265) = 2.126, *d* = 0.26, p = 0.035), as compared to the more Difficult poems (Difficult poem, Easy versus Difficult font: M_difference_ = -0.10, t(244) = -0.547, *d* = -0.07, p = 0.585).

All results for the third dependent variable (‘Perceived Structure Clarity’) are presented in Table 3C and illustrated in Figs 2C, 3C and 4C We observed that the perceived clarity of the structure of easy poems was higher than that of more difficult poems (main effect of Poem Difficulty). Again, and crucially, there was a statistically significant Poem Difficulty x Font Readability interaction. For the Easy poems there was a statistically significant difference with Easy font leading to higher perceived Structure Clarity as compared to Difficult font (Easy poem, Easy versus Difficult font: M_difference_ = 0.48, t(265) = 3.115, *d* = 0.38, p = 0.002). For the Difficult poems this effect was numerically reversed, albeit not statistically significantly so (Difficult poem, Easy versus Difficult font: M_difference_ = -0.28, t(244) = -1.504, *d* = -0.19, p = 0.134).

Finally, the results for the fourth dependent variable (‘Perceived Topic Clarity’) are presented in Table 3D and illustrated in Figs 2D, 3D and 4D. We observed that easy poems were perceived to have higher topic clarity than more difficult poems (main effect of Poem Difficulty). We did not observe a statistically significant Poem Difficulty x Font Readability interaction (p = 0.116). Nor were there statistically significant differences in the planned comparisons (Easy poem, Easy versus Difficult font: M_difference_ = 0.20, t(265) = 1.178, *d* = 0.14, p = 0.240; Difficult poem, Easy versus Difficult font: M_difference_ = -0.14, t(244) = -0.693, *d* = -0.09, p = 0.489).

The covariates Age and the answer to the question ‘Do you consider yourself to be a poetry lover?’ showed considerable spread (Age: see above; Poetry Lover: Mean = 2.95; median = 3; range = 1–7; SD = 1.52, IQR = 2). These covariates explained significant portions of variance (see Table 3A–3D). Both Age and whether participants considered themselves to be poetry lovers had a positive relationship with each of the four dependent variables. That is, older participants on average rated the poems higher on the four dependent variables as compared to younger participants. Similarly, those who consider themselves poetry lovers rated the poems on average higher than those who consider themselves less of a poetry lover.

In sum, we observe that Font Readability has a differential influence on three of the four dependent variables related to the poem reading experience for Easy compared to Difficult poems (Font Readability x Poem Difficulty interaction). This effect is mainly driven by a decrease in General Liking, Perceived Flow, and Perceived Structure Clarity when Easy poems are presented in a difficult to read font.

As an exploratory analysis for general interest, we computed correlations between all the dependent variables. As is clear from Table 4, all dependent variables were positively correlated with each other. This indicates that poems that were for instance scored high on General Liking also tended to be scored high on Perceived Flow. While some of these dependent variables could theoretically be assumed to be separable, this analysis suggests that in participants’ actual scorings of poems they are related.

**Table 4:** Correlations between dependent variables. There were sizeable positive correlations between how participants scored the poems on the four main dependent variables (General Liking, Flow, Structure Clarity, Topic Clarity). All of these variables correlate negatively with perceived difficulty.

Correlations between dependent variables						
		**General Liking**	**Flow**	**Structure**	**Topic**	**Difficulty**
General Liking	Spearman’s rho	—				
	p-value	—				
Flow	Spearman’s rho	0.649	—			
	p-value	< .001	—			
Structure	Spearman’s rho	0.490	0.576	—		
	p-value	< .001	< .001	—		
Topic	Spearman’s rho	0.494	0.525	0.465	—	
	p-value	< .001	< .001	< .001	—	
Difficulty	Spearman’s rho	-0.378	-0.447	-0.316	-0.430	—
	p-value	< .001	< .001	< .001	< .001	—

## Discussion

In this study we investigated how Font Readability influences the subjective experience of poetry. Specifically, we were interested in how Easy and Difficult poems could be differently influenced by the readability of the font they are presented in. The starting point of our investigation was that font readability would influence processing fluency and would change the subjective experience of poetry (e.g. [1]). Subjective experience was measured using four dependent variables, each tapping into a different aspect of how a poem can be perceived. Participants rated their general liking of the poems, and the perceived structural and conceptual fluency of the poems.

We hypothesized that while a less readable font would lead to lower scores on these dependent variables for Easy poems, the reversed pattern would be observed for Difficult poems. This hypothesis was partially confirmed. Statistically speaking we did find evidence for a differential effect of Font Readability for the Easy and Difficult poems. That is, we found that there was a Font Readability x Poem Difficulty interaction effect for three out of the four dependent variables. This interaction effect was however largely driven by an Easy versus Difficult font difference for the Easy poems. Easy poems overall got rated lower when presented in a difficult to read font as compared to when presented in an easy to read font. This is an interesting finding in itself as it suggests that breaking the natural flow of reading has an influence on the way people experience poetry. The effect sizes of this effect (for three out of four dependent variables) are small to medium (Cohen’s *d* of 0.40, 0.26, 0.38). The Font Readability x Poem Difficulty effect was not observed for the dependent variable Topic Clarity. Given the strong correlations between the dependent variables and given that the F-value for the interaction term related to Topic Clarity was sizeable (F(1,490) = 2.48), we refrain from interpreting the absence of a statistically significant effect as proof for Topic Clarity being quantitatively different from the other variables.

Another part of our prediction was however not confirmed. Directly comparing Easy versus Difficult font for the Difficult poems did not reveal evidence for the hypothesized beneficial effect of difficult to read font for difficult poems. We did not find evidence for a reverse effect of font readability on easy versus difficult poems. In summary, Easy poems are ‘hindered’ by a difficult to read font, but no effects of Font Readability were observed for Difficult poems. A body of literature shows that disfluency can have a beneficial effect on the processing of difficult stimuli (see overview in [9]). A hypothesized mechanism for the beneficial effect of disfluency would be that reduced fluency leads to deeper, more careful processing. From our results it seems that this did not happen for the difficult poems that our participants read. Based on the data that we acquired little can be said about why disfluency did not have a beneficial effect on the difficult poems. We for instance did not measure reading times to corroborate that the manipulation of font did lead to increased processing of the poems (see discussion below).

Our findings indicate that manipulating the ease of processing at early levels of reading (via font readability) can influence the aesthetic appreciation of poems. This evidence lines up with previous literature showing effects of font readability on processing [17]; (see [9] for overview), and adds font readability to a list of features influencing language reception [2–7,31,38,39]. An interesting issue for follow-up studies would be to investigate the effect of written (e.g. font) or phonological form on the perceived *emotional content* of the poems. Several lines of work indicate that the perceived emotional content of a poem is at least partially explained by the phonological and / or prosodic content of poems [31,40–45].

Another hypothesis we had was that the interaction effect between Font Readability and Poem Difficulty would be different for perceived structural and conceptual fluency. Since structural and conceptual fluency differed between the poems of the three experiments, we expected that the Font Readability x Poem difficulty interaction would be different across experiments, depending on which dependent variable was tested. This was not borne out in our data. We did not find any statistical interactions with the factor Experiment, despite the fact that structural and conceptual difficulty differed between the poems that were used as stimuli in the different experiments. This result can be taken as evidence in favor of a ‘general’ fluency effect, that is, an effect of any kind of fluency, no matter what the main source of the fluency is. Indeed, in their influential overview of a diverse literature, Alter and Oppenheimer conclude that fluency has a general effect on processing ([8], see also [33]). The current findings are in line with such an account. Future research is needed to confirm or disconfirm the unity of fluency for poetry reception.

An alternative explanation of the lack of distinct effect for structural and conceptual fluency is that our survey questions were not sensitive enough to pick up a difference. It is hard to assess whether this is the case or not. What we can say is that the dependent variables correlate highly with each other, but that this correlation is not extremely high either. The dependent variables were chosen to measure presumably separable aspects of the subjective experience of poetry. We asked participants to rate the overall liking, the perceived flow, the perceived topic clarity, and the perceived structure of a poem. Despite the fact that these measures theoretically tap into different aspects of poetry reception (unsurprisingly mapping onto structural and conceptual fluency), they were strongly correlated. The pattern of scorings on each of the variables was very comparable across conditions. Perhaps participants used general ‘liking’ as the basis for their assessment of all aspects of the poems. The apparent similarity with which the dependent variables were treated raises the question what ‘naive theory’ participants employed when making their judgements of the poem. Naive theories are (implicit) ideas that people have on why they experience fluency or disfluency [33,46,47]. In the case of our stimuli, it may be that the effect of the hard to read font on the experience of easy poems is a reflection of the naive theory that something which is difficult to read is less beautiful. A related effect of participants’ naive theories could be that when they encounter a mismatch with their (implicit) expectation, this leads to decreased appreciation. One could argue that next to its influence on readability, the font manipulation also led to a violation of expectation. After all, it is uncommon (although not unheard of, see introduction) to read poetry in a font as peculiar as Mistral. Indeed, current psychological models about art reception presume that a work of art is matched against an expected form format [48]. Expectation violation could have added to the effect in our study, but we believe that it is not a full alternative for readability as the main driving factor. After all, we measured how readable participants considered the Arial and Mistral font and observed that the latter scored much lower on readability.

Menninghaus and colleagues coined the ‘handicap principle’, meaning that rhetorical features such as rhyme and meter will induce a cognitive handicap, hindering more semantic (what we called ‘conceptual’ here) processing [5]. We did not observe that poems high on rhetorical features such as rhyme and meter were considered less easy to understand semantically. One reason may be that in the present materials it was not the case that structural or conceptual fluency features were absent versus present. The materials were much better characterized as containing a graded mix of rhetorical features. This may have obscured any potential ‘handicap’ effects. A related issue is whether higher levels of different kinds of fluency by necessity increase appreciation. In the framework of ‘parallelistic diction’ it has been suggested that a combination of rhetorical features surpasses appreciation of poems which contain only one (or at least fewer) rhetorical features [7,39]. Our findings are supportive of this, although it is difficult to make the case decisively since we did not manipulate the degree of rhetorical features in our materials.

A shortcoming of the current study is that for practical reasons we did not measure reading time (note that we did measure how long participants took to read the poems for the data that were acquired in the online questionnaire. Since this is only the case for a small proportion of our sample (12%) we decided not to report those results). It would be interesting for future work to investigate whether participants read the more difficult to read font more slowly or not. Wallot and Menninghaus [7] found that reading times for proverbs with meter were longer than for those without meter. That is, stimuli with higher structural fluency were read more slowly. This is in line with evidence showing that too much processing time towards the specifics of a narrative can be detrimental for the experience of a narrative. For instance, de Vries and colleagues showed that participants that had shortest eye gaze durations when reading metaphors in a literary text, reported to be the most absorbed in the narrative. Moreover, these participants liked the story more than participants who did spend more time on the metaphors [49]. Jacobs in his model of poetic reading associates foregrounding with deepened processing, as reflected in slower reading [29,30]. It is an intriguing thought that foregrounding as an example of disfluency does not necessarily lead to slower or ‘deeper’ processing. Future research should investigate the influence of processing time on the subjective experience of poetry more fully. Note that when we speak about ‘readers’ we base our conclusions on a sample which was not biased for their knowledge of poetry. Indeed, the average score on the question how much participants considered themselves to be poetry lovers was around 3 (on a 1–7 scale). It is likely that results would look different for more trained poetry readers.

In terms of generalizability it should be clear–but is worthwhile stressing anyhow–that with only six poems used as stimulus materials, presented in only two different fonts, the generalizability of our findings to other poems and fonts remains to be assessed in future research. As far as the poems are concerned we note that we sampled broadly–but not systematically—from Dutch poetry, and that there is previous literature showing partially comparable findings in other languages, with other materials. Still, we want to make clear that we cannot claim our results to be generalizable based on this limited sample of poems and fonts.

An aspect of our study which is nice (even if it also complicates interpretation somewhat) is that we used existing, unmodified poems from Dutch literature. It means that we measured people’s reactions to poems in their ultimate, published state. This is in contrast to a sizeable body of psycholinguistic literature in which stimuli are created by the researcher (see discussion in [50]). Poets consciously and subconsciously combine rhetorical features when writing their poetry, and we kept this intact in the present study. Finally, we measured appreciation in a more diverse sample than is typically done in experimental psychology. We hope our study can be inspirational for sampling cognition ‘in the wild’ using original, unmodified stimuli [50–52].
